# Plant-soil feedback responses to drought are species-specific and only marginally predicted by root traits

**DOI:** 10.1007/s11104-024-07049-z

**Published:** 2024-11-07

**Authors:** Eileen Enderle, Fangbin Hou, Leonardo Hinojosa, Hidde Kottman, Nigâr Kasirga, Franciska T. de Vries

**Affiliations:** Institute for Biodiversity and Ecosystem Dynamics, University of Amsterdam, Amsterdam, The Netherlands

**Keywords:** Drought, Grassland, Plant-soil feedback, Root exudation, Root traits, Soil microbes

## Abstract

**Background and aims:**

The increasing occurrence of extreme drought events under climate change alters the composition and functioning of plant communities worldwide. Drought-induced changes in plant-soil feedback (PSF), reciprocal effects on fitness between plants and their associated soil microbial communities, are one mechanism through which these changes in vegetation occur, but they remain difficult to predict. Because of their direct link to rhizosphere microbial communities, we expect root traits to predict drought-induced PSF shifts.

**Methods:**

In the conditioning phase of a greenhouse experiment, we subjected 12 common grassland species to drought. In the feedback phase, all species were grown under ambient conditions with their own microbial inoculum. Their growth was compared to growth in sterile soil to assess total PSF or soil inoculated with microbes from three other species to assess specific PSF. We used root traits to predict PSF under drought and ambient conditions.

**Results:**

Drought altered the magnitude and direction of PSF in a quarter of the species, which was consistent between total and specific PSF. Total PSF was best predicted by the first axis of the root trait space (high specific root length to high root diameter and root nitrogen content) and was not responsive to drought. Specific PSF was weakly predicted by root traits and changed in response to drought.

**Conclusion:**

Our results show that drought can modify the feedbacks between plants and their microbial communities with implications for vegetation dynamics. Root traits have limited capacity to predict these shifts, but can predict PSF of the total microbial community independent of drought.

**Supplementary Information:**

The online version contains supplementary material available at 10.1007/s11104-024-07049-z.

## Introduction

Climate change is increasing the frequency and intensity of extreme drought events in many regions of the world (Spinoni et al. 2018, IPCC Chapter 4,2023), which can persistently alter plant community composition (Hoover et al. 2014; Liu et al. 2018a; Stampfli et al. 2018). These shifts are the result of direct effects of drought on plants, but also of altered plant-microbial interactions in the soil (Angulo et al. 2022). Feedbacks between plants and their associated soil microbiota (plant-soil feedback, PSF; Bever et al. 1997) are widely recognized as an important driver of many processes that determine plant species distribution (Klironomos 2002; van der Putten et al. 2013; Lekberg et al. 2018). Both positive PSF, characterised by a facilitative microbial community, and negative PSF, characterised by a harmful microbial community, are important for maintaining plant species coexistence, preventing dominance and determining successional pathways (Bever 2003; Kardol et al. 2006; Goossens et al. 2023). Being able to predict the direction and magnitude of PSF across different species and environments is essential for understanding its role in plant community dynamics (van der Putten et al. 2016).

PSF is driven by direct beneficial or harmful effects of soil microbes on plants, or indirectly through decomposition of litter inputs (Wardle et al. 2004; Bennett and Klironomos 2019; Veen et al. 2019). Plant functional groups and growth strategies explain some variation in PSF: grasses, which tend to attract many pathogens, generally experience the most negative PSF, forbs experience negative to neutral PSF, whereas legumes, which are more dependent on beneficial microbes, often experience the most positive PSF (Kulmatiski et al. 2008; Cortois et al. 2016; Martorell et al. 2021; Lozano et al. 2022). These broad categories, however, are limited in their ability to explain the high variation in PSF across species, experimental settings and environments (Smith-Ramesh and Reynolds 2017; de Long et al. 2019).

Belowground traits may improve the prediction of PSF as roots form the direct interface between plants and soil microbes. Two important trait axes have been identified, which define the root economics space (Bergmann et al. 2020). The conservation axis classifies plants by their resource acquisition strategy, separating slow-growing, well-defended plants with a high root tissue density (RTD) from fast-growing plants with a high root nitrogen content (RNC) and low defence (Bergmann et al. 2020). The collaboration axis classifies plants based on their dependence on soil microorganisms, distinguishing ‘do-it-yourself’ strategists with a high specific root length (SRL) from species with a high root diameter (RD) which outsource their resource acquisition to symbionts (Bergmann et al. 2020). Rutten and Allan (2023) showed that the location and distance of the ‘home’ species and the ‘away’ species in the root economics space were strong predictors of PSF, which was generally more negative in slow-growing species. Cortois et al. (2016) found a negative relationship of PSF with SRL of the home species and a positive correlation with mycorrhizal colonisation, showing that investment in collaboration with beneficial microbes results in more positive PSF. Beyond these well-established root traits, root exudation is increasingly recognised as a functional trait that shapes plant-microbial interactions, microbial activity (Zhalnina et al. 2018; Guyonnet et al. 2018; Williams et al. 2021b), and consequently PSF (Hu et al. 2018; Steinauer et al. 2023). However, to fully understand the interplay of root traits, root exudation and PSF we need more studies assessing these relationships on a broad range of species, ecosystems and environmental pressures (de Vries et al. 2016).

There is growing evidence that climate change, and drought in particular, can alter PSF (Pugnaire et al. 2019; Beals et al. 2020; Hassan et al. 2022b). It has been proposed that PSF becomes more positive under moderate drought due to decreased pathogen pressure, because many fungal and bacterial pathogens depend on moist conditions (Pandey et al. 2015; Delavaux et al. 2021), and increased benefits from cooperation with mutualists such as mycorrhizal fungi or plant growth-promoting bacteria (PGPB) (Rubin et al. 2017; Jia et al. 2020; de Vries et al. 2023). Under severe drought and the recovery thereof, however, beneficial interactions often get disrupted and PSF may become less positive for slow-growing, collaborative species (de Vries et al. 2023). The limited number of experimental studies assessing drought effects on PSF are not consistent in their findings. In some cases, there is a tendency for negative PSF being alleviated under drought and PSF on average becoming more positive (Fry et al. 2018; Snyder and Harmon-Threatt 2019; Martorell et al. 2021; Xi et al. 2022). In other experiments, PSF turns more negative under drought (Dudenhöffer et al. 2022), which was connected to the abundance of parasitic nematodes (Hassan et al. 2022a) or a disruption of both pathogenic and symbiotic relationships with soil fungi (Lozano et al. 2022). However, only a portion of these differences can be explained by functional group effects (Hassan et al. 2021; Martorell et al. 2021). Recent evidence shows that more negative PSF under drought was linked to a shift in its prediction by root traits (Lozano et al. 2022). Thus, including the root economics space in explaining PSF responses to drought may increase the accuracy of our predictions and our general understanding of plant-microbial interactions under drought.

Here, we aimed to test the impact of severe drought on PSF across functional groups and to determine to what extent the root economics space can predict PSF under ambient and drought conditions. We distinguished between total PSF (growth in home soil vs. sterile soil) and specific PSF (growth in home soil vs. away soil conditioned by another species), and hypothesised that (I) drought relieves negative PSF in grasses and forbs by reducing pathogen pressure, and weakens positive PSF in legumes by disrupting beneficial interactions; further (II) we expected more positive total PSF in collaborative, slow-growing species with a large RD, high specific exudation rate (SER) and RTD and the most negative total PSF in less collaborative, fast-growing species with high RNC and SRL; (III) we expected the location of both home and away species on the root economic space to predict specific PSF, but the collaboration axis to be more important; (IV) we hypothesised that drought would decrease the strength of the positive relationship between PSF and collaborative traits by weakening the impact of beneficial microbes.

We tested these hypotheses in a two-stage greenhouse experiment with 12 common grassland species of three functional groups. In the conditioning phase, all species were grown with or without an extreme drought treatment and left to recover for one week. These soils were used as inocula in the feedback phase to test drought legacy effects on total and specific PSF in the same species under ambient conditions. We then linked PSF in droughted and non-droughted soils to root traits measured in both home and away species at the end of the conditioning phase.

## Materials and methods

### Plant and soil origin

**Fig. 1:**
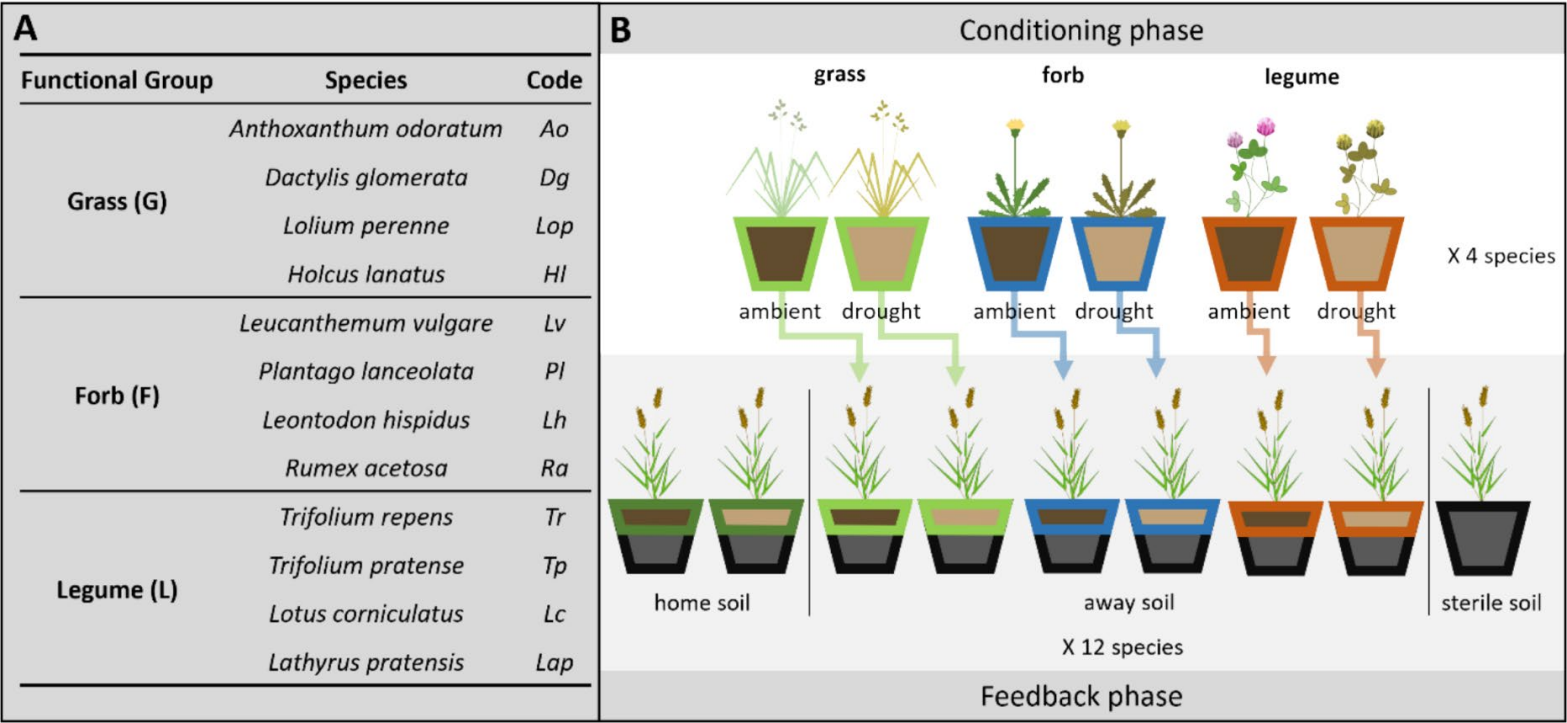
Experimental setup. **A** Full names, functional groups and abbreviations of all 12 plant species. **B** Setup of conditioning and feedback phase. During the conditioning phase, four species of each functional group were grown under drought and ambient conditions. The soil was used as inoculum in the feedback phase, in which five replicates of all species were grown in nine different soil mixtures: Sterile soil inoculated with 30% of their home soil (drought and ambient), sterile soil inoculated with away soil from one other grass, forb and legume (drought and ambient), and 100% sterile soil

A sandy loam soil (SOM = 5.5 ± 1.5%, pH = 7.75 ± 0.24, soil moisture = 16.7%), excavated from a nearby grassland, was purchased from Den Ouden Group (Netherlands). Soil was sieved through a 4 mm mesh and stored at 4 °C until the start of the experiment. Twelve common grassland species (Fig. 1) belonging to three plant functional groups (four grasses, forbs and legumes) were selected to represent a range of growth strategies and drought tolerance levels. Seeds were purchased from a local company (Cruydt-Hoeck, NL).

### Conditioning phase

#### Experimental setup

The first phase of the experiment was set up in the greenhouse in summer 2021, with a 16 h:8 h day-night cycle and an average temperature of 21 °C. Seeds of *Lathyrus pratensis* were pretreated at 4 °C for four weeks and then germinated with all other seeds for two weeks in unsterilised soil. Individual seedlings were transplanted into 648 ml pots (SOPARCO, 9 × 9 × 10 cm) with 600 g moist, unsterilised soil. All plants grew for two months under ambient conditions and were watered at least every other day to a water-holding capacity (WHC) of 60% (18% gravimetric soil moisture). Then, half of the pots were subjected to a two-week treatment of drought (WHC: 20%) followed by a week of recovery (WHC: 60%), following de Vries et al. (2019). To be able to completely separate soil-microbial mediated effects from direct drought effects and to focus on drought legacy effects which keep affecting plant community dynamics after the drought has ended, we chose to impose a drought in the conditioning phase, but not in the feedback phase. Each treatment was repeated five times in a randomized block design (three functional groups x four species x two treatments x five blocks = 120 pots). At the end of the recovery phase, soil was carefully shaken off the root systems, mixed, sieved through a 2 mm mesh and stored separately at 4 °C until use as inoculum. Soil chemical properties were measured in water extracts (DOC and pH) or 1 M KCl extracts (plant-available nutrients; Table S2). Dissolved and plant-available nutrients (NH_4_^+^, NO_3_^−^, PO_4_^3−^) were measured in the AutoAnalyzer (SAN^++^ SYSTEM, SKZLAR), dissolved organic carbon (DOC) in the TOC-V CPH (SHIMADZU) and pH in a multi-channel analyser (CONSORT C831).

#### Root trait analysis

Each root system was carefully washed and all debris removed with tweezers. To prepare for root exudate collection, plants were transferred to 100 ml grass SCHOTT bottles (DURAN®) containing 100 ml hydroponics solution (100 g soil/L; Williams et al. 2021a). Bottles with plants were placed in a climate chamber under 16 h light at 22 °C and 8 h night at 20 °C for one week and aerated with compressed air (1 bubble/s). The solution was exchanged once after three days. Root exudates were collected by shaking the root system of the intact plant in 100 ml sterile milli-Q for 2 h at 60 rpm on ice and vacuum filtration through 2 µm filter paper (10,401,712, Whatman™, UK). Total root exudate carbon per plant (exudate C) was measured as DOC in the TOC-V CPH (SHIMADZU). Root systems were scanned with WINRHIZO® ROOT ANALYSIS software to determine average root diameter (RD), root volume and total root length. Roots were dried at 70 °C for 48 h and weighed on a four-decimal scale. SRL was determined by dividing total root length by root biomass, RTD by dividing root biomass by root volume, root dry matter content (RDMC) by dividing root dry biomass by fresh biomass and SER by dividing exudate C by root biomass. Dry roots were milled in a plant mill (ZM 200 Retsch®) and analysed for RNC and root carbon content (RCC) in the elemental analyser (vario EL CUBE, Elementar).

### Feedback phase

In the feedback phase, the same 12 species were grown in the greenhouse for seven weeks in spring 2022. To calculate two types of PSF, each species grew in nine different soil types (Fig. 1): 100% sterile soil (sterile soil) or 70% sterile soil mixed with 30% inoculum from either soil conditioned by the same species (home soil) or from soil conditioned by a randomly selected grass, forb and legume (away soil; Table S1). Home and away soil came from either droughted (drought) or well-watered plants (ambient). This inoculation approach was chosen to reduce nutrient effects through sterilisation and from nutrient depletion in the conditioning phase. All treatments were replicated five times in a randomized block design according to the blocks in the conditioning phase (three functional groups x four species x nine soil treatments x five blocks = 540 pots).

The sterile background soil came from the same batch as the conditioned soil and was sterilized by gamma irradiation at 30 kGy/min (STERIS, Ede, NL). The conditioned soil was left to re-acclimatise to room temperature for 48 h and mixed with the sterile soil in a ratio of 3:7 according to dry mass. The moisture in all soil mixtures was elevated to 60% WHC and 250 g moist soil was filled in 228 ml planting pots (Modiform, 7 × 7 × 8 cm) and placed in petri dishes to avoid cross-contamination. Seeds of all species were surface-sterilized with 40 ml 1% chlorine-based household bleach for 1 min, then washed with 40 ml milli-Q five times in 50 ml centrifuge tubes. Multiple seeds were sown in 5 mm deep holes according to their germination rate and excess seedlings were removed after germination. Pots without germination received a seedling transfer from surplus seeds germinated on filter paper. Plants were watered with rainwater at least every three days by softly sprinkling every pot for three seconds and regularly weeded.

Seven weeks after sowing aboveground biomass was harvested by cutting at soil level. Belowground biomass was carefully washed and freed from debris with tweezers. Plant biomass was oven-dried at 70 C for 48 h and weighed on a four-decimal scale (Table 1).

**Table 1:** Replication statement

Variables of interest	Scale of inference	Scale at which the factor of interest is applied	Number of replicates at the appropriate scale
biomass root traits soil properties total PSF	species	plant/pot	5 plants per species and treatment
biomass root traits soil properties total PSF	plant functional group (FG)	plant/pot	4 species per FG, each including 5 individual plants (= 20 pants per FG and treatment)
specific PSF	species pair	plant community	36 species pairs per treatment, consisting of 12 species

### Data analysis

#### Plant-soil feedback

Plant-soil feedback (PSF) was calculated with total dry biomass in home soil compared to either sterile soil (total PSF; feedback of the entire microbial community) or away soil from each of the three other species (specific PSF; feedback of only the species-specific part of the microbial community) as follows:

$$\begin{array}{l}{PSF(total)}_{A}=ln \left(\frac{{biomass A}_{a}}{{biomass A}_{st}}\right)\\\\ {PSF(specific)}_{A}=ln \left(\frac{{biomass A}_{a}}{{biomass A}_{b}}\right)\end{array}$$

Here, *A* stands for the focal species; *a* for its own conditioned soil (home soil); *st* for sterile soil and *b* for soil conditioned by another species *B* (away soil). Feedbacks were calculated according to replicates to limit the impact of block effects.

#### Statistical analysis

All statistical analyses were performed in R (version 4.4.1) with Rstudio (version 2024.04.1). All models were tested for normal distribution of their residuals and homoscedasticity by visual inspection of density plots, QQ plots and residual plots. Outliers were checked for anomalies during the data collection phase, for instance, early death or anomaly in the phenotype, and respective data points were excluded. If not mentioned otherwise all analyses were based on linear mixed models using the ‘lme4’ package (Bates et al. 2015) with experimental block as random effect.

Effects of drought and plant identity and their interaction on plant biomass and PSF were determined with a type III analysis of variance (ANOVA). In separate models, plant identity either referred to home species or functional group. In functional group models home species was accounted for as random effect. Total and specific PSF were analysed in separate models with away species as a random effect in the latter. Drought effects on PSF within groups were identified with pairwise Tukey tests.

To describe the root economic space we performed a principal component analysis (PCA) with root trait data collected at the end of the conditioning phase, including SRL, RD, RNC, RCC, C:N ratio, RDMC, RTD, SER and exudate C. As the resulting PCA did not entirely represent the root economics space defined by Bergmann (2020), we refer to it as root trait space from hereon and all following analyses were based on its first two axes (PC1 and PC2). Predicted loadings of total and specific PSF were added to the PCA as supplementary variables and therefore did not contribute to determining the principal components. Their direction and length represent their relationship with the other variables in this trait space. The effect of plant functional group, drought and their interaction on the location of a plant on PC1 and PC2 was tested by ANOVA and pairwise Tukey tests, with species as a random effect.

We assessed the PCA location of plants on the root trait space as a predictor for PSF separately for total and specific PSF. Total PSF was modelled with the location of the home species on PC1 and PC2 and their interaction with drought as fixed effects. The relationship between the root trait space and specific PSF was modelled based on the location of both home and away species on PC1 and PC2, their interaction with each other and with drought. As we did not find an interaction between both PCA axes in predicting PSF in model comparisons based on AIC (Akaike information criterion), this interaction was removed from the model (AIC_interaction_ = 417.78, AIC_separate_ = 399.78). To account for variation within each species pair, a random effect of away species nested in home species was included.

The contribution of individual root traits to explaining variation in PSF was analysed in a backwards model selection process. First, global models including all analysed root traits were tested for collinearity of predictors. C:N ratio was excluded in both models for total and specific PSF due to high correlation with RNC. Then, interactions with drought and for specific PSF with the trait data from the away species were added to the models, respectively. Using the ‘step’ function from the ‘LmerTest’ package (Kuznetsova et al. 2017), in each elimination step the least-contributing predictor was removed until only significant predictors and interactions remained in the final model (elimination threshold *P* = 0.1). Away species nested in home species was added as random effect in the model for specific PSF.

## Results

### Plant functional group and drought effects on PSF

**Fig. 2:**
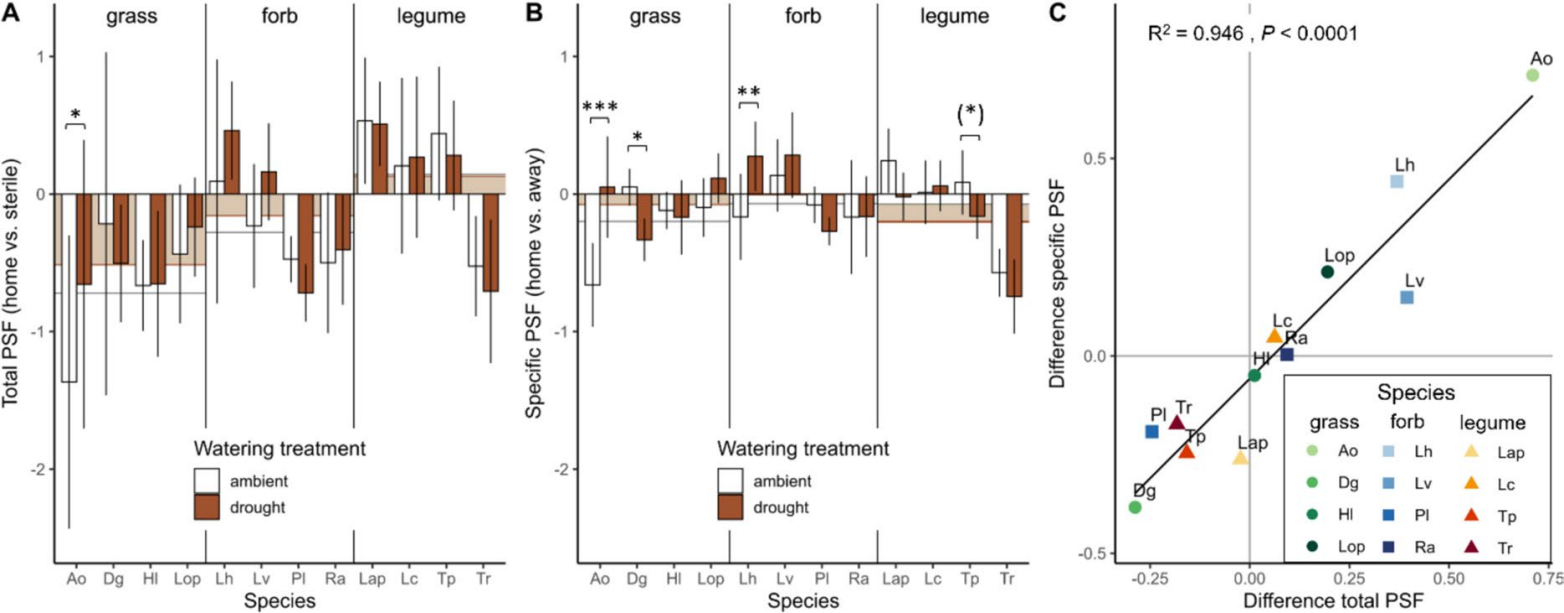
**A** Total and **B** specific PSF under drought (red) and ambient (white) conditions across 12 species. Error bars represent 95% confidence intervals of the mean, indicating PSF different from 0 if they do not overlap with the x-axis. Asterisks show significant differences between ambient and drought at *P* < (*) 0.1, * 0.05, ** 0.01, *** 0.001 (pairwise Tukey). White and red boxes in the background represent functional group means of ambient and drought treatments. **C** Change of PSF under drought compared to ambient conditions (PSF_drought_ – PSF_ambient_) for both PSF types. Points represent the mean difference per species. Pearson correlation coefficient and respective *P*-value are shown. Species abbreviations see Fig. 1

Overall, most plants produced more biomass in sterile soil and in away soil than in their home soil (Fig. S1), resulting in overall negative total PSF (– 0.23 ± 0.63; Fig. 2A) and specific PSF (– 0.11 ± 0.48, Fig. 2B), respectively. Plant functional group impacted total PSF, but not specific PSF, in its direction and strength (Fig. 2A; Table 2): grasses experienced the most negative total PSF (– 0.62 ± 0.60, Fig. 2A), forbs weak negative feedback (–0.22 ± 0.51, Fig. 2A) and legumes slightly positive feedback (0.14 ± 0.55; Fig. 2A). However, the variation between species within each functional group was high. Total and specific PSF were correlated (Fig. S2), but specific PSF was overall more negative and weaker than total PSF.

**Table 2:** Type III ANOVA output of plant identity (species or functional group) and drought effects on total and specific PSF. Models with functional group were corrected for species as a random effect. Presented statistics are degrees of freedom (DF), Chi-squared (χ^2^), their respective *P*-values and adjusted R^2^ for fixed and random effects

Total PSF						
Term	PI = functional group			PI = species		
	DF	χ^2^	*P*-value	DF	χ^2^	*P*-value
Plant identity (PI)	**2**	**7.716**	**0.021**	**11**	**78.489**	** < 0.0001**
Watering treatment (WT)	1	2.064	0.151	**1**	**6.776**	**0.009**
PI: WT	2	1.899	0.387	11	11.705	0.386
	R^2^ adj. fixed: 0.22 random: 0.32			R^2^ adj. fixed: 0.52 random: 0.01		
Specific PSF						
Term	PI = functional group			PI = species		
	DF	χ^2^	*P*-value	DF	χ^2^	*P*-value
Plant identity (PI)	2	0.802	0.670	**11**	**62.981**	** < 0.0001**
Watering treatment (WT)	1	2.441	0.118	**1**	**21.117**	** < 0.0001**
PI: WT	**2**	**6.894**	**0.032**	**11**	**45.722**	** < 0.0001**
	R^2^ adj. fixed: 0.01 random: 0.24			R^2^ adj. fixed: 0.23 random: 0.06		

The drought response of total and specific feedback was highly correlated (Fig. 2C, R^2^ = 0.946, *P* < 0.0001). In all species except *H. lanatus*, the direction of PSF change under drought corresponded between the two feedback types and in the majority of those the magnitude of change was equal (Fig. 2C). Drought shifted total PSF to be on average less negative from – 0.28 ± 0.66 to – 0.17 ± 0.59, but in individual species this was only significant for *A. odoratum* (Fig. 2A; Table 2). The effect of drought on specific PSF depended on species and functional group identity in its magnitude and direction (Fig. 2B; Table 2): in grasses and forbs negative specific PSF was on average reduced or even neutralised, whereas slightly negative feedback in legumes was intensified (Fig. 2B). In three out of the 12 species a shift of specific PSF was found in response to drought (Tukey; *P* < 0.05), including two grass species and one forb.

### The root trait space as a predictor for PSF under drought

**Fig. 3:**
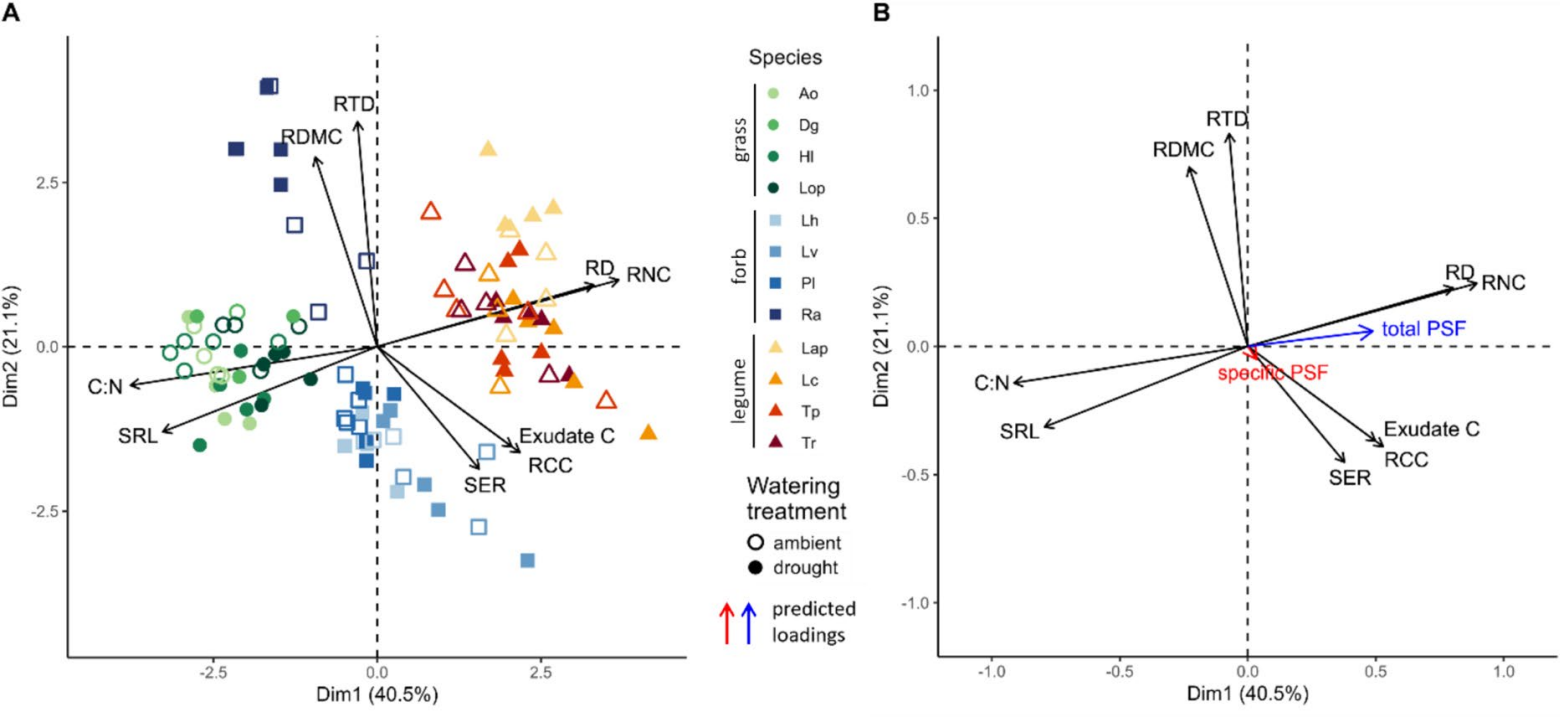
Principal component analysis (PCA) of the first two dimensions of the root trait space. **A** Individual plants of all species under drought (filled symbols) and ambient (open symbols) conditions. Shape represents the functional group (circles = grasses, squares = forbs, triangles = legumes), and colour shade represents the species. **B** Predicted loadings of the supplementary variables total PSF (blue) and specific PSF (red) based on the PCA of the root traits of the home species. RD = root diameter, RDMC = root dry matter content, RNC = root nitrogen content, RTD = root tissue density, SER = specific exudation rate, SRL = specific root length. Species abbreviations see Fig. 1

With root trait data from the conditioning phase (Table S3), we established a root trait space (Fig. 3A) that explained 40.5% of the overall variation by its first axis (PC1) and 21.1% of variation by its second axis (PC2). PC1 divided plants with a high SRL and high root C:N ratio from plants with a high RD and high RNC. The second axis ran from high SER, exudate C and RCC towards high RTD and RDMC. PC1 clearly separated plant functional groups (Fig. 3A, Table S4, ANOVA, *P* < 0.0001): grasses generally had the highest SRL and C:N ratio, forbs the lowest RTD and RDMC and legumes the highest RNC and RD. Drought effects on the position of species on both axes of the trait space depended on their functional group (Table S4, ANOVA, *P* < 0.05). On average, drought shifted grasses and legumes towards a lower RD and a higher SRL on PC1, grasses towards a higher RTD on PC2, while forbs were not affected (Fig. S3).

**Fig. 4:**
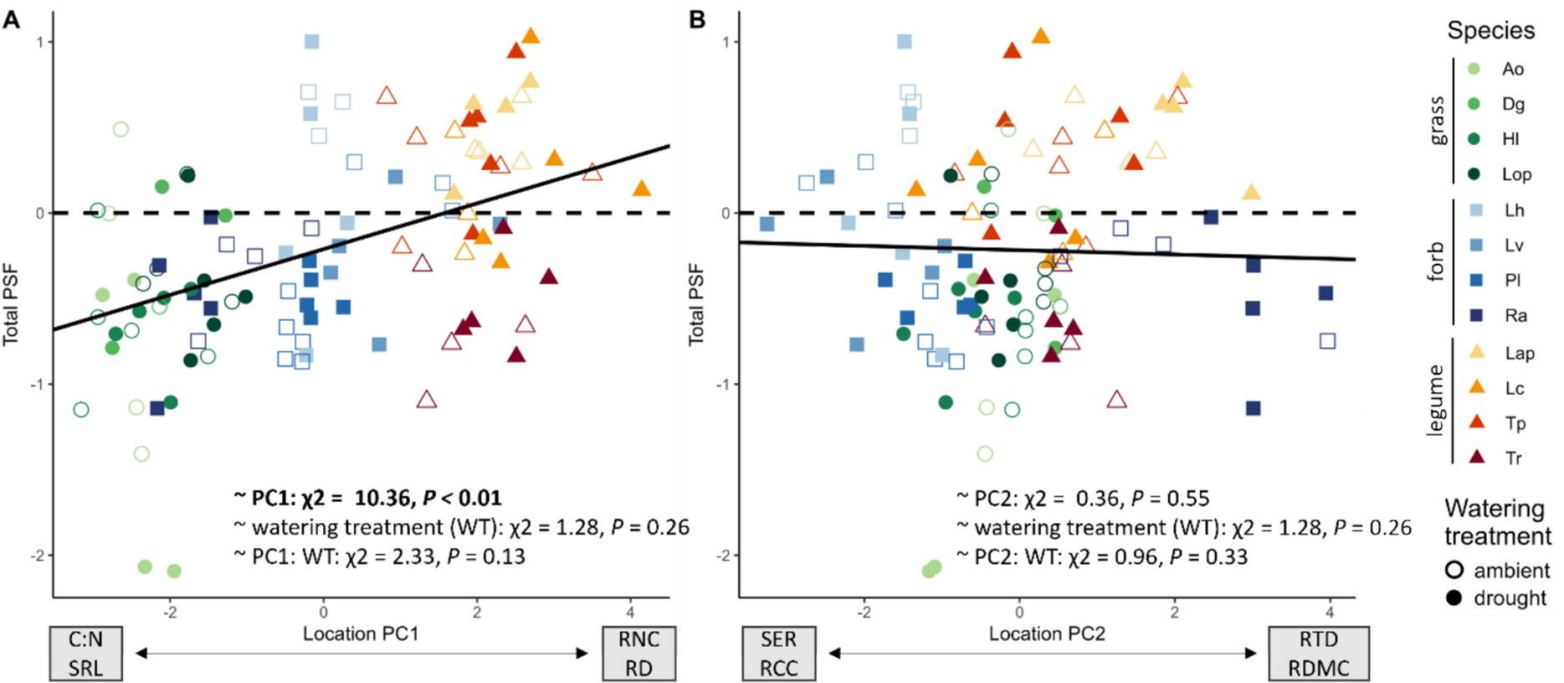
Relationship between the position of individual plants on **A** PC1 and **B** PC2 of the root trait space and their total PSF under droughted (filled symbols) or ambient (open symbols) conditions. Black lines represent model estimates, based on linear mixed effects models accounting for species and block as random effects. Species abbreviations see Fig. 1

Total PSF was predicted by a plant’s position on PC1 but not PC2 (Figs. 3B and 4; Table S5): total PSF was more positive for plants with a high RD and RNC and more negative for plants with a high SRL and C:N ratio (Fig. 4A). This was linked to the position of the functional groups along that same axis: grasses with a higher SRL and C:N ratio experienced more negative total PSF and legumes with a higher RD and a high RNC experienced more positive total PSF (Fig. 4A). Overall, the PCA location of a plant on the root trait space explained 29% of the total variation in total PSF (Table S5). Drought in the conditioning phase did not affect the prediction of total PSF by either axis of the root trait space (Fig. 4).

**Fig. 5:**
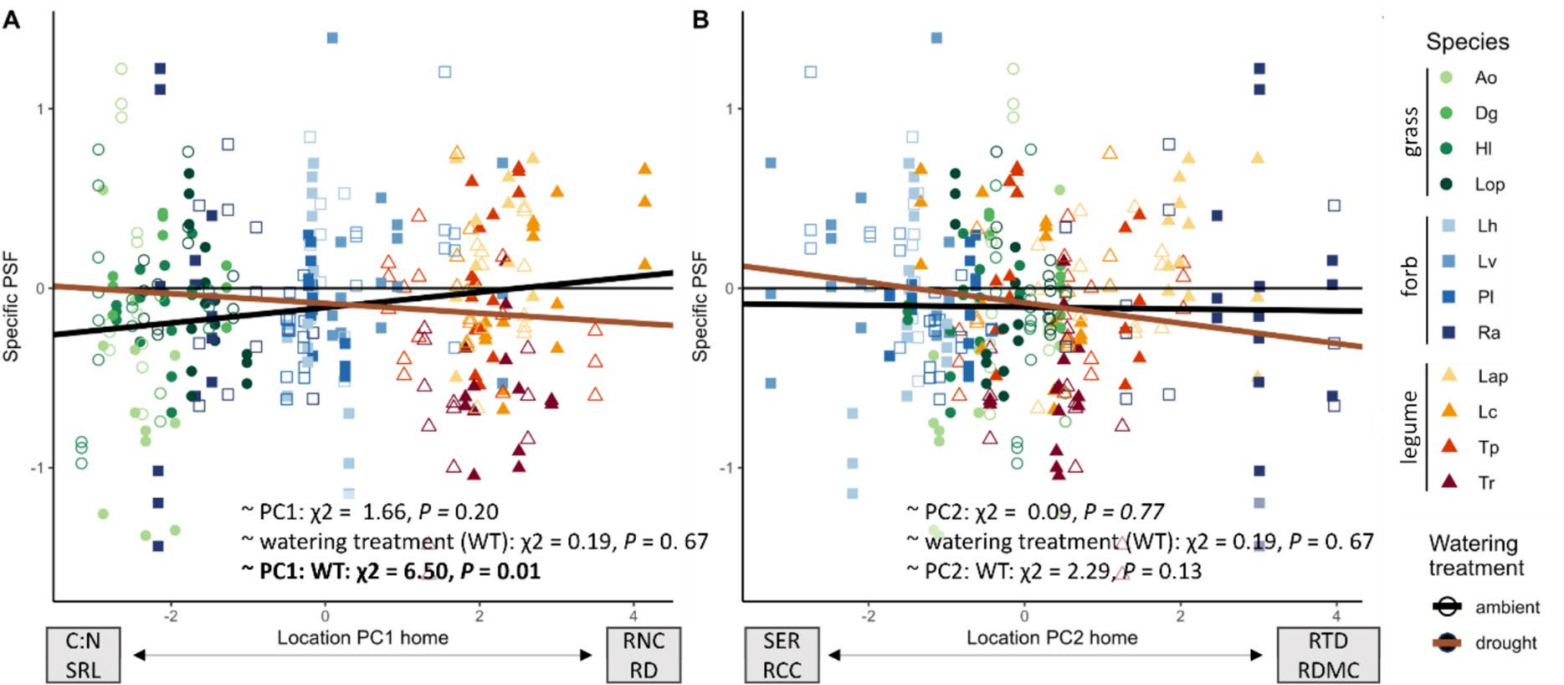
Relationship between the location of the home species on **A** PC1 and **B** PC2 of the root trait space and specific PSF under droughted (full symbols) and ambient (open symbols) conditions. Lines represent model estimates for drought (red) and ambient (black) based on a linear mixed effects model accounting for home species, away species and block as random effects. Species abbreviations see Fig. 1

Using the location of both home and away species on both trait axes, we evaluated how well the root trait space could predict specific PSF and whether that changed in response to drought. Only the location of the home species on PC1 contributed significantly to explaining variation in specific PSF and this relationship changed under drought (Fig. 5, Table S5). Under ambient conditions, plants with a high SRL and C:N ratio experienced more negative PSF, whereas plants with a high RD and RNC experienced more positive PSF. This reversed with drought, resulting in more negative PSF in plants with a high RD and RNC. A similar pattern was found for PC2, however, this was not significant at *P* = 0.1 (Fig. 5B, Table S5). Our model explained 21% of all variation in specific PSF, of which, however, only 1% was explained by the fixed terms (Table S5).

### Individual root traits as predictors of total and specific PSF

**Fig. 6:**
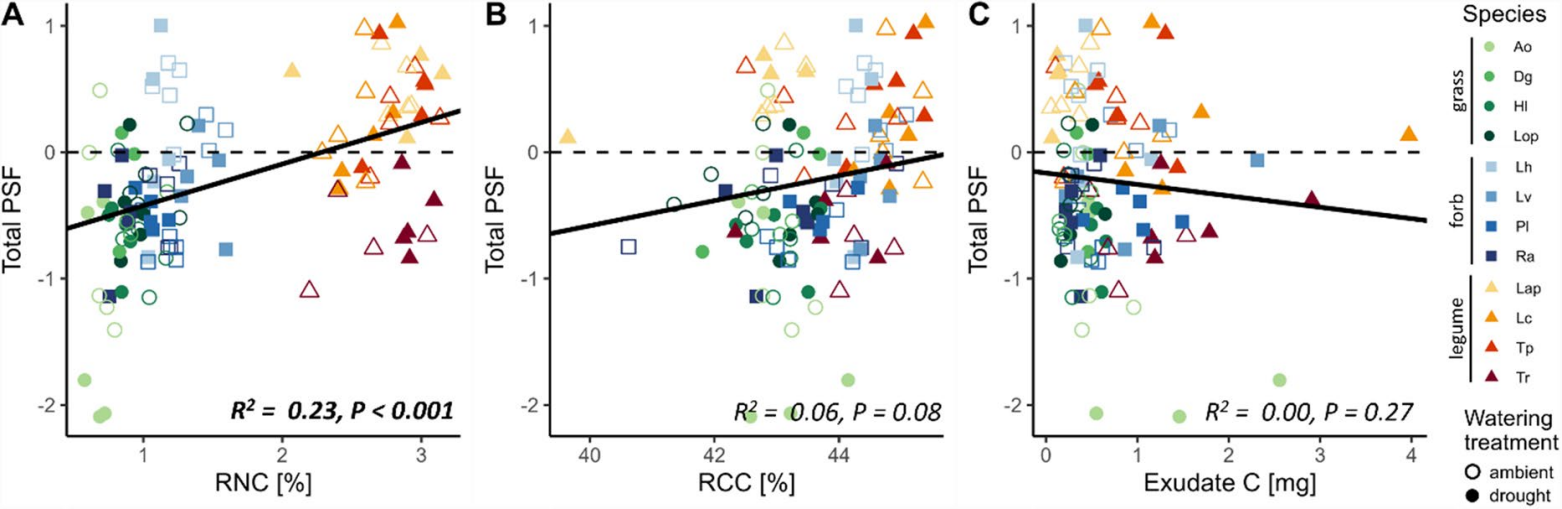
Relationship between total PSF and the root traits selected as predictors. Circles represent ambient conditions and squares drought in the conditioning phase. Shape represents the functional group (circles = grasses, squares = forbs, triangles = legumes) and the colour shade represents the species. Black lines represent model estimates from mixed effects models of the respective trait, accounting for block, species and drought as random effects; corresponding R^2^ and *P*-values are presented in each panel. RNC = root nitrogen content, RCC = root carbon content. Species abbreviations see Fig. 1

To determine which root traits drove these relationships between the root trait space and total and specific PSF, we determined the strongest predictors and their interactions with drought by backwards model selection (Table 3). RNC, exudate C and RCC together explained 30% of variation in total PSF, which was unaffected by drought (Table 3). RNC and RCC were positively related to total PSF, whereas total root exudate C was negatively related to total PSF (Fig. 6). Specific PSF was predicted by a combination of six traits and/or their interaction with drought (Table 3). The strongest predictors were SER and RDMC in a three-way interaction of the trait data from home and away plant and drought (Table 3, Fig. S6). For RD and exudate C, the trait value of the away species was more important for predicting specific PSF than that of the home species. SRL and RCC did not contribute to explaining specific PSF. Overall, the fixed terms of the model explained approximately 8% of the total variation in specific PSF (Table 3).

**Table 3:**
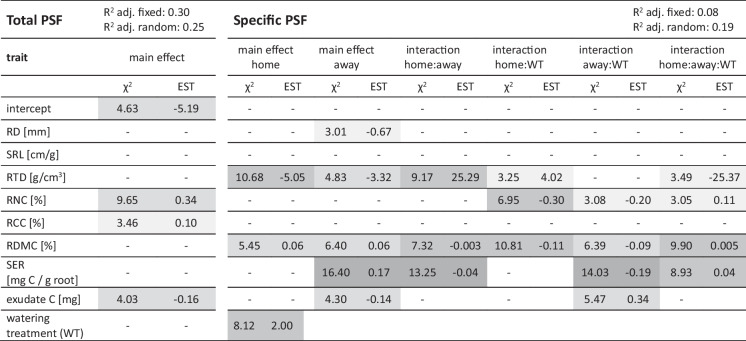
Model outputs for predicting total and specific PSF based on root traits of the home and away species and their interaction with drought (soil treatment, ST). Models were obtained through stepwise backwards selection of linear mixed effects models at an elimination threshold of *P* < 0.1. Presented statistics are their respective Chi-squared (χ^2^) and model estimates (EST). Grey shades represent *P*-values below 0.1 (lightest grey), 0.05 (light grey), 0.01 (dark grey) and 0.001 (darkest grey). *RD* root diameter, *SRL *specific root length, *RTD* root tissue density, *RNC* root nitrogen content, *RCC* root carbon content, *RDMC* root dry matter content, *SER* specific exudation rate

## Discussion

Here, we assessed how severe drought and its soil legacy impacts PSF and how this is linked to plant functional group and the root trait space. We present evidence that drought-induced shifts in PSF are species-specific, largely independent of functional group, and that total PSF (home vs. sterile soil) and specific PSF (home vs. away soil) responses to drought are highly similar. We found that the main axis of the root trait space, running from high SRL and C:N ratio towards high RD and RNC, explained more variation in total PSF than specific PSF, suggesting that belowground economic strategies are good indicators for the general effect of a plant’s soil microbial community on its own growth. Specific PSF was better predicted by a combination of individual root traits of both the home and away species, including root exudation traits. The relationship between specific PSF, but not total PSF, and root traits changed in response to drought, stressing the importance of including climate change factors when assessing PSF and its impact on vegetation dynamics.

### Effects of drought on PSF

We found that drought shifted PSF in only three out of 12 species, indicating that the microbial groups driving PSF were only affected in some species or that positive and negative microbial impacts on plant growth balanced each other out in many species. There is great variability in PSF responses to drought across studies, ranging from no effect on total PSF (Wilschut and van Kleunen 2021) to species-dependent drought effects (Hassan et al. 2021; Lozano et al. 2022), to consistent drought effects across species (Kaisermann et al. 2017; Xi et al. 2022). These differences between studies may be the consequence of the large variation of experimental setups and drought intensity, duration and application during different experimental phases (de Vries et al. 2023). As drought was only applied during the conditioning phase, our experiment represents PSF dynamics in the recovery phase after the extreme drought has ended. Here, species identity was more important than functional group in determining whether and how PSF responded to drought (Table 2), which was in contrast to our first hypothesis and other studies that found different PSF responses to drought between functional groups (Hassan et al. 2021; Martorell et al. 2021). As hypothesized, drought weakened negative PSF in grasses and forbs, which could be the consequence of a general disruption of plant-microbial interactions, for instance, reduced pathogen pressure (Desprez-Loustau et al. 2006; Choudhary and Senthil-Kumar 2024). In grasslands, however, most studies find increased relative abundances of fungal pathogens in response to drought (de Oliveira et al. 2020; Francioli et al. 2020), or taxa-dependent drought responses (Ochoa-Hueso et al. 2018).

Our finding that PSF responses to drought were inconsistent within functional groups, but highly correlated between both PSF types and more pronounced in specific PSF (Fig. 3) suggests that the underlying mechanisms may be species-specific. Specific PSF is generally associated with specialist pathogens and mutualists (Klironomos 2002; Bever 2002), but more research is recognising a role for both generalist and specialist microbes in determining specific PSF (Semchenko et al. 2022; Xi et al. 2022). Both root-associated fungal and bacterial communities are determined by species identity of the host plant (Bever 2002; Fitzpatrick et al. 2018) and their drought responses can be species-specific (Kaisermann et al. 2017). Especially fungal pathogen communities have been shown to have distinct host ranges (Ampt et al. 2022) and species-specific responses to drought (Francioli et al. 2020), making them likely drivers of drought-induced shifts in PSF. Challenging that, Lozano et al. (2022) showed that drought increased the influence of saprotroph and eradicated the impact of mutualist or pathogen communities on specific PSF. This highlights the importance of microbial community data in future research for identifying the role of different microbial groups in driving PSF shifts under drought.

### Prediction of PSF by the root trait space

We show that the first two axes of the root trait space explain a significant amount of variation in PSF, although this variation ranges from 21% for total PSF to only less than 1% for specific PSF (Table S5). This suggests that the effects of the whole rhizosphere microbial community are better predicted by general resource acquisition strategies than species-specific plant-microbial interactions, which might be better predicted by qualitative traits such as root exudate composition. Against our expectations, drought did not affect the relationship between root traits and total PSF indicating that neither trait plasticity under drought nor microbial drought responses disrupted the interactions with the soil microbial community as a whole. The primary trait axis (from high SRL and C:N ratio to high RD and RNC; Fig. 3) which largely corresponds with the collaboration gradient (Bergmann et al. 2020), was more important than the second axis (from high exudate C and SER to high RTD and RDMC) in predicting both types of PSF. This confirms our hypothesis that more collaborative plants with high RD, such as legumes, generally profited from their associated microbes whereas plants with a “do-it-yourself” resource acquisition strategy and a high SRL, here predominantly grasses, generally suffered from their associated microbial community (Kulmatiski et al. 2008; Cortois et al. 2016; Semchenko et al. 2022). Most likely, this is based on legumes benefitting more from collaboration with rhizobia and mycorrhizal fungi, while grasses are more vulnerable to pathogens (Sweeney et al. 2021; Ampt et al. 2022). Contrary to our expectation, the second trait axis was not important for determining PSF. Unlike other studies, which also included woody species (Bergmann et al. 2020; Rutten and Allan 2023; Liang et al. 2023), we could not identify the typical conservation gradient, as RNC corresponded with the collaboration gradient because it was correlated with RD, as a result of those traits both being high in legumes (similar to Sweeney et al. 2021). This challenges if the root economics space accurately represents root trait distribution in grassland species when including legumes.

### Prediction of PSF by individual root traits

When considering individual root traits instead of trait axes the amount of explained variation increased to 30% for total PSF and 8% for specific PSF (Table 3). Nevertheless, the large amount of residual variation means there must be other processes driving PSF that are not covered by the measured traits. Total PSF was positively correlated with RNC, likely based on N-fixation in legumes, and with RCC, which may be linked to the accumulation of structural carbohydrates such as lignin, which can enhance defence against pathogens by increasing the physical barrier and thus reducing negative PSF (Liu et al. 2018b). For both types of PSF, root exudation traits played a significant role in predicting PSF. Distinct profiles and temporal patterns of root exudation may thus be one mechanism by which plant species accumulate unique microbial communities that in turn affect their own performance, as shown in other studies (Hu et al. 2018; Zhao et al. 2021; Steinauer et al. 2023). During and after drought, species-specific changes in these root exudation profiles could lead to shifts in PSF, which is supported by our observation that SER and exudate C predicted specific PSF differently under drought conditions. Accordingly, previous research shows that drought-induced changes in the quantity and quality of rhizodeposits impact microbial community composition and activity (Preece and Peñuelas 2016; Gargallo-Garriga et al. 2018) and possibly these qualitative differences are more important than the quantity of exudation in determining specific PSF. Further, RTD and RDMC, traits associated with resource conservation, contributed to explaining specific PSF (Table 3), suggesting a role of the conservation gradient in determining patterns of PSF as previously reported (Baxendale et al. 2014; Rutten and Allan 2023), for example by increasing the abundance of saprotroph and mutualist fungi (Lozano et al. 2021). Confirming our third hypothesis, for five out of the six traits that predicted specific PSF, trait data from both the home and away species contributed significantly to the model. Accordingly, specific PSF was determined by different root traits and microbial groups in conspecific and heterospecific soils before (Semchenko et al. 2018; Lozano et al. 2022). This underlines how traits of neighbouring plants can impact the fitness of individual plants through microbial feedback. Greenhouse experiments are essential for identifying these mechanisms, but their relevance needs to be tested in field experiments (Beals et al. 2020).

## Conclusion

We demonstrate that drought can shift PSF, but that the magnitude and direction of change varies across species and the underlying drivers seem to differ between species. Such high variation in PSF response to drought may destabilise mechanisms for species coexistence and maintaining diversity in plant communities, and it is key to test these mechanisms and their relevance for vegetation dynamics in the field. Only to a limited extent we were able to link these drought effects on PSF to the root trait space, exposing the need for further research to uncover the underlying mechanisms. Independent of drought, our results demonstrate the suitability of root traits, in particular those related to the collaboration gradient, to predict feedbacks of the total microbial community, but their limitations when it comes to feedbacks from species-specific microbes. Assessing specific mechanisms of plant-microbial interactions in more detail, such as root exudate and microbial community composition, but also eco-evolutionary dynamics between plant and their associated microbes, may improve our prediction of PSF and the implications for vegetation dynamics. Overall, this study advances our understanding of the role of rhizosphere plant-microbial interactions in mediating plant community response to extreme climatic events.

## Supplementary Information

Below is the link to the electronic supplementary material.

Supplementary file1 (DOCX 7.96 MB)

## Data Availability

Upon publication, all corresponding data will be published on Figshare (10.21942/uva.26348590), according to the data policy of the journal.
