# Supplementary material for: Plant-soil feedback responses to drought are species-specific and only marginally predicted by root traits
Source: Plant Soil. 2024 Nov 7;511(1-2):1205–20. doi: 10.1007/s11104-024-07049-z (PMC12227364; doi:10.1007/s11104-024-07049-z)
Supplement: Supplementary file 1 — Supplementary file1 (DOCX 7.96 MB) [file 11104_2024_7049_MOESM1_ESM.docx]

Supplementary Information

**Table S1:** Plant species–inoculum combinations used in the feedback phase for the three functional groups. These combinations were randomly selected and used for both ambient and drought inoculum.

**Table S2**: Soil chemical parameters at the end of the conditioning phase of soil conditioned by all species under ambient and drought conditions (mean ± standard deviation). DOC = dissolved organic carbon,

TDN = total dissolved nitrogen.

**Table S3.** Root trait values at the end of the conditioning phase for all species under ambient and drought conditions (mean ± standard deviation). RTD = root tissue density, RD = root diameter, SRL = specific root length, RNC = root nitrogen content, RCC = root carbon content, RDMC = root dry matter content,

SER = specific exudation rate.

**Table S4:** Type III ANOVA output of the location on the two first axes of the root trait space according to plant functional group and drought. Presented statistics are degrees of freedom (DF), Chi-squared (χ^2^) and their respective *P*-values. All values are rounded to three decimals. Values for PC1 are square-root-transformed to meet the test assumptions.

**Table S5:** Type III ANOVA results of total and specific PSF according to the location of home (and away) species on the first two axes of the root trait space (PC1, PC2) and how this is affected by drought (watering treatment, WT). Presented statistics are degrees of freedom (DF), Chi-squared (χ2), their respective

*P*-values and the model formula.

**Figure S1**: Plant aboveground (green) and belowground (brown) dry biomass across all soils at the end of the feedback phase. Black error bars within the columns represent standard errors for above- and belowground biomass separately. Grey error bars above the columns represent the standard error for the total biomass. Within each species, groups that have no letter in common are significantly different in their total biomass (Tukey, P < 0.05); ns = not significant. Watering treatments describe the type of inoculum added: A = ambient, D = drought. The title above two columns describes the plant origin of that inoculum.

**Figure S2:** Correlation between total and specific PSF. Open symbols represent ambient and filled symbols drought species means. Pearson correlation coefficient and the respective *P*-value are shown on top. Species abbreviations see Fig. 1.

**Figure S3:** Boxplots showing the location of plants on the first two axes of the root trait space dependent on their functional group and watering treatment. Ambient plants are presented in white and droughted plants in red. Asterisks represent differences between drought and ambient conditions within each functional group at *P* < 0.05 (Tukey).

**Figure S4:** Mean specific PSF for each species with ambient (white) and droughted (red) soil inoculum from each conditioning functional group. Error bars represent 95% confidence intervals of the mean. Error bars not crossing the x-axis indicate PSF significant difference from 0 (one-sample t-test). *P*-values indicate significant differences between ambient and drought at (*) *P* < 0.1, * *P* < 0.05, ** *P* < 0.01, *** *P* < 0.001 (Tukey).

**Figure S5**: Relationship between total PSF and root traits that were not selected in the model selection process. Open symbols represent ambient conditions and closed symbols drought in the conditioning phase. Shape represents the functional group (circles = grasses, squares = forbs, triangles = legumes) and colour shade represents the species. Black lines represent model estimates from mixed effects models, accounting for block, species and drought effects. RD = root diameter, SRL = specific root length,

RTD = root tissue density, RDMC = root dry matter content, SER = specific exudation rate. Species abbreviations see Fig. 1.

**Figure S6**: Relationship between specific PSF and root traits of home and away species and under ambient (A – D) and drought (E – H) conditions. Presented traits showed a significant 3-way interaction effects between home trait, away trait and watering treatment on specific PSF.

**Table S1**

| Focal plant species | Soil inoculum grass | Soil inoculum forb | Soil inoculum legume |
| --- | --- | --- | --- |
| *Anthoxanthum odoratum* | *Lolium perenne* | *Rumex acetosa* | *Lotus corniculatus* |
| *Dactylis glomerata* | *Holcus lanatus* | *Leucanthemum vulgare* | *Trifolium pratense* |
| *Holcus lanatus* | *Anthoxanthum odoratum* | *Leontodon hispidus* | *Lathyrus pratensis* |
| *Lolium perenne* | *Dactylis glomerata* | *Plantago lanceolata* | *Trifolium repens* |
| *Leontodon hispidus* | *Anthoxanthum odoratum* | *Leucanthemum vulgare* | *Lotus corniculatus* |
| *Leucanthemum vulgare* | *Lolium perenne* | *Plantago lanceolata* | *Lathyrus pratensis* |
| *Rumex acetosa* | *Holcus lanatus* | *Leontodon hispidus* | *Trifolium repens* |
| *Plantago lanceolata* | *Dactylis glomerata* | *Rumex acetosa* | *Trifolium pratense* |
| *Lathyrus pratensis* | *Lolium perenne* | *Leontodon hispidus* | *Trifolium repens* |
| *Lotus corniculatus* | *Holcus lanatus* | *Leucanthemum vulgare* | *Trifolium pratense* |
| *Trifolium pratense* | *Anthoxanthum odoratum* | *Rumex acetosa* | *Lathyrus pratensis* |
| *Trifolium repens* | *Dactylis glomerata* | *Plantago lanceolata* | *Lotus corniculatus* |

**Table S2**

| Species | Watering  treatment | pH | DOC [μg/g] | Dissolved  NO3 [μg/g] | Dissolved  NH4 [μg/g] | TDN [μg/g] | Plant-available NO3 [μg/g] | Plant-available NH4 [μg/g] | Plant-available PO4 [μg/g] |
| --- | --- | --- | --- | --- | --- | --- | --- | --- | --- |
| *Ao* | ambient | 7.72 ± 0.41 | 22.20 ± 2.93 | 0.50 ± 0.42 | 0.085 ± 0.040 | 2.33 ± 0.43 | 0.905 ± 0.571 | 0.506 ± 0.130 | 0.275 ± 0.043 |
|  | drought | 7.76 ± 0.39 | 25.05 ± 3.50 | 1.70 ± 1.02 | 0.119 ± 0.039 | 3.71 ± 1.08 | 2.412 ± 1.155 | 0.275 ± 0.023 | 0.372 ± 0.047 |
| *Dg* | ambient | 7.73 ± 0.40 | 22.85 ± 3.42 | 0.88 ± 0.43 | 0.067 ± 0.016 | 2.80 ± 0.40 | 1.214 ± 0.317 | 0.298 ± 0.012 | 0.216 ± 0.034 |
|  | drought | 7.77 ± 0.35 | 24.25 ± 3.71 | 2.09 ± 0.32 | 0.070 ± 0.033 | 4.70 ± 1.54 | 2.552 ± 0.467 | 0.287 ± 0.032 | 0.319 ± 0.037 |
| *Hl* | ambient | 7.79 ± 0.30 | 21.35 ± 2.40 | 0.91 ± 0.36 | 0.060 ± 0.044 | 2.62 ± 0.62 | 1.569 ± 0.319 | 0.214 ± 0.100 | 0.289 ± 0.075 |
|  | drought | 7.82 ± 0.30 | 24.39 ± 4.32 | 2.68 ± 1.46 | 0.079 ± 0.041 | 4.61 ± 1.50 | 5.734 ± 2.307 | 0.235 ± 0.089 | 0.500 ± 0.051 |
| *Lop* | ambient | 7.82 ± 0.33 | 23.94 ± 3.46 | 0.68 ± 0.11 | 0.081 ± 0.052 | 2.71 ± 0.20 | 1.271 ± 0.300 | 0.297 ± 0.066 | 0.309 ± 0.051 |
|  | drought | 7.83 ± 0.38 | 24.17 ± 4.96 | 1.73 ± 0.92 | 0.092 ± 0.051 | 3.74 ± 0.93 | 3.508 ± 1.355 | 0.168 ± 0.041 | 0.576 ± 0.053 |
| *Lh* | ambient | 7.80 ± 0.23 | 21.63 ± 3.29 | 0.58 ± 0.36 | 0.077 ± 0.046 | 2.21 ± 0.47 | 1.395 ± 0.986 | 0.234 ± 0.051 | 0.270 ± 0.040 |
|  | drought | 7.80 ± 0.34 | 23.68 ± 3.45 | 1.71 ± 0.28 | 0.103 ± 0.045 | 3.86 ± 0.84 | 4.201 ± 1.108 | 0.167 ± 0.152 | 0.360 ± 0.035 |
| *Lv* | ambient | 7.79 ± 0.31 | 21.57 ± 3.76 | 0.86 ± 0.34 | 0.077 ± 0.041 | 2.42 ± 0.27 | 1.551 ± 0.716 | 0.291 ± 0.120 | 0.241 ± 0.087 |
|  | drought | 7.82 ± 0.42 | 23.54 ± 3.94 | 1.68 ± 0.21 | 0.100 ± 0.028 | 4.46 ± 1.02 | 3.863 ± 0.802 | 0.332 ± 0.151 | 0.367 ± 0.119 |
| *Ra* | ambient | 7.70 ± 0.25 | 22.51 ± 1.55 | 0.63 ± 0.29 | 0.081 ± 0.043 | 2.45 ± 0.48 | 2.281 ± 0.809 | 0.163 ± 0.112 | 0.237 ± 0.096 |
|  | drought | 7.75 ± 0.28 | 24.60 ± 1.94 | 1.82 ± 1.02 | 0.077 ± 0.038 | 3.81 ± 1.11 | 4.958 ± 2.268 | 0.154 ± 0.074 | 0.401 ± 0.086 |
| *Pl* | ambient | 7.82 ± 0.29 | 24.94 ± 3.99 | 0.53 ± 0.37 | 0.048 ± 0.018 | 2.35 ± 0.40 | 0.849 ± 0.364 | 0.229 ± 0.067 | 0.283 ± 0.028 |
|  | drought | 7.77 ± 0.34 | 25.24 ± 3.18 | 1.76 ± 0.67 | 0.084 ± 0.042 | 4.02 ± 1.44 | 3.889 ± 0.888 | 0.322 ± 0.130 | 0.505 ± 0.082 |
| *Lap* | ambient | 7.76 ± 0.23 | 23.14 ± 4.91 | 4.62 ± 2.82 | 0.070 ± 0.013 | 6.80 ± 3.16 | 6.517 ± 2.845 | 0.209 ± 0.167 | 0.506 ± 0.048 |
|  | drought | 7.82 ± 0.34 | 22.41 ± 5.82 | 6.95 ± 1.91 | 0.105 ± 0.035 | 9.22 ± 2.06 | 9.718 ± 2.941 | 0.169 ± 0.118 | 0.478 ± 0.081 |
| *Lc* | ambient | 7.78 ± 0.12 | 27.84 ± 7.10 | 0.49 ± 0.42 | 0.128 ± 0.039 | 2.68 ± 0.51 | 1.695 ± 0.971 | 0.132 ± 0.111 | 0.177 ± 0.041 |
|  | drought | 7.81 ± 0.24 | 26.20 ± 4.96 | 5.34 ± 4.74 | 0.062 ± 0.017 | 7.60 ± 4.72 | 8.537 ± 5.692 | 0.163 ± 0.126 | 0.273 ± 0.067 |
| *Tp* | ambient | 7.80 ± 0.16 | 25.85 ± 4.52 | 1.01 ± 0.44 | 0.050 ± 0.019 | 3.04 ± 0.30 | 2.608 ± 1.164 | 0.173 ± 0.063 | 0.106 ± 0.011 |
|  | drought | 7.75 ± 0.25 | 25.27 ± 4.49 | 6.40 ± 3.68 | 0.104 ± 0.040 | 8.78 ± 3.88 | 10.431 ± 6.035 | 0.181 ± 0.083 | 0.254 ± 0.043 |
| *Tr* | ambient | 7.76 ± 0.14 | 25.57 ± 4.72 | 1.43 ± 0.48 | 0.149 ± 0.061 | 3.65 ± 0.66 | 4.116 ± 1.411 | 0.193 ± 0.120 | 0.404 ± 0.118 |
|  | drought | 7.77 ± 0.22 | 24.77 ± 4.37 | 9.36 ± 4.89 | 0.070 ± 0.015 | 11.38 ± 4.88 | 8.372 ± 4.358 | 0.158 ± 0.084 | 0.182 ± 0.066 |

**Table S3**

| Species | Watering  treatment | Root dry biomass [g] | Root:  shoot Ratio | RTD [g/cm^3^] | RD [mm] | SRL [cm/g] | RNC [%] | RCC [%] | C:N ratio | RDMC [%] | Exudate C [mg] | SER [mg/g] |
| --- | --- | --- | --- | --- | --- | --- | --- | --- | --- | --- | --- | --- |
| *Ao* | ambient | 0.349 ± 0.031 | 0.403 ± 0.102 | 0.149 ± 0.024 | 0.131 ± 0.005 | 507 ± 54 | 0.66 ± 0.07 | 43.02 ± 0.70 | 65.78 ± 7.65 | 21.03 ± 3.62 | 1.10 ± 0.91 | 3.06 ± 2.39 |
|  | drought | 0.273 ± 0.094 | 0.418 ± 0.070 | 0.165 ± 0.028 | 0.129 ± 0.005 | 477 ± 82 | 0.70 ± 0.07 | 43.05 ± 0.37 | 61.68 ± 5.68 | 23.59 ± 2.30 | 0.52 ± 0.25 | 1.89 ± 0.45 |
| *Dg* | ambient | 0.294 ± 0.093 | 0.356 ± 0.176 | 0.167 ± 0.033 | 0.131 ± 0.014 | 471 ± 129 | 0.85 ± 0.06 | 42.12 ± 2.37 | 49.79 ± 4.85 | 24.49 ± 2.76 | 0.39 ± 0.08 | 1.50 ± 0.75 |
|  | drought | 0.251 ± 0.071 | 0.388 ± 0.144 | 0.176 ± 0.027 | 0.125 ± 0.023 | 490 ± 111 | 0.93 ± 0.14 | 42.68 ± 0.74 | 46.41 ± 5.79 | 24.75 ± 2.17 | 0.23 ± 0.15 | 1.09 ± 1.04 |
| *Hl* | ambient | 0.298 ± 0.085 | 0.276 ± 0.117 | 0.140 ± 0.024 | 0.127 ± 0.014 | 611 ± 250 | 0.83 ± 0.07 | 43.01 ± 0.60 | 52.09 ± 4.64 | 19.77 ± 1.98 | 0.55 ± 0.09 | 2.10 ± 1.13 |
|  | drought | 0.188 ± 0.049 | 0.260 ± 0.106 | 0.144 ± 0.023 | 0.109 ± 0.010 | 785 ± 222 | 0.97 ± 0.14 | 43.06 ± 0.29 | 45.23 ± 6.53 | 26.62 ± 3.29 | 0.26 ± 0.14 | 1.43 ± 0.64 |
| *Lop* | ambient | 0.283 ± 0.051 | 0.394 ± 0.084 | 0.165 ± 0.017 | 0.146 ± 0.009 | 371 ± 84 | 0.92 ± 0.07 | 43.35 ± 0.28 | 47.48 ± 3.53 | 17.30 ± 1.82 | 0.36 ± 0.21 | 1.37 ± 1.02 |
|  | drought | 0.200 ± 0.068 | 0.462 ± 0.216 | 0.155 ± 0.023 | 0.125 ± 0.018 | 552 ± 137 | 1.09 ± 0.19 | 42.27 ± 0.62 | 39.57 ± 6.31 | 22.78 ± 2.26 | 0.28 ± 0.04 | 1.59 ± 0.73 |
| *Lh* | ambient | 0.331 ± 0.075 | 0.517 ± 0.153 | 0.108 ± 0.015 | 0.221 ± 0.015 | 250 ± 54 | 1.10 ± 0.06 | 44.18 ± 0.25 | 40.31 ± 2.21 | 10.79 ± 0.42 | 0.57 ± 0.33 | 1.98 ± 1.74 |
|  | drought | 0.244 ± 0.064 | 0.762 ± 0.393 | 0.105 ± 0.022 | 0.238 ± 0.017 | 227 ± 60 | 1.18 ± 0.07 | 44.35 ± 0.17 | 37.62 ± 2.29 | 10.82 ± 0.43 | 0.36 ± 0.14 | 1.47 ± 0.41 |
| *Lv* | ambient | 0.233 ± 0.024 | 0.336 ± 0.102 | 0.109 ± 0.018 | 0.230 ± 0.016 | 232 ± 64 | 1.42 ± 0.14 | 44.55 ± 0.21 | 31.53 ± 3.12 | 10.97 ± 0.29 | 0.99 ± 0.84 | 4.37 ± 3.67 |
|  | drought | 0.197 ± 0.086 | 0.359 ± 0.112 | 0.111 ± 0.025 | 0.246 ± 0.060 | 230 ± 129 | 1.51 ± 0.08 | 44.83 ± 0.22 | 29.83 ± 1.58 | 12.56 ± 1.70 | 1.01 ± 0.32 | 6.07 ± 3.29 |
| *Ra* | ambient | 0.748 ± 0.127 | 0.885 ± 0.120 | 0.384 ± 0.063 | 0.161 ± 0.018 | 131 ± 21 | 0.83 ± 0.08 | 42.95 ± 0.55 | 52.39 ± 4.83 | 30.75 ± 2.97 | 0.35 ± 0.15 | 0.46 ± 0.16 |
|  | drought | 0.428 ± 0.075 | 0.947 ± 0.146 | 0.312 ± 0.092 | 0.154 ± 0.009 | 187 ± 65 | 1.17 ± 0.08 | 43.34 ± 1.69 | 37.15 ± 2.62 | 24.26 ± 1.71 | 0.52 ± 0.38 | 1.36 ± 1.22 |
| *Pl* | ambient | 0.317 ± 0.082 | 0.319 ± 0.085 | 0.143 ± 0.033 | 0.218 ± 0.015 | 200 ± 58 | 1.06 ± 0.09 | 43.68 ± 0.41 | 41.43 ± 3.68 | 14.66 ± 3.83 | 0.97 ± 0.39 | 2.98 ± 0.82 |
|  | drought | 0.229 ± 0.034 | 0.410 ± 0.126 | 0.112 ± 0.012 | 0.218 ± 0.017 | 247 ± 54 | 1.14 ± 0.12 | 43.44 ± 0.61 | 38.51 ± 4.54 | 13.91 ± 3.26 | 0.45 ± 0.17 | 1.99 ± 0.80 |
| *Lap* | ambient | 0.171 ± 0.137 | 0.357 ± 0.169 | 0.194 ± 0.032 | 0.565 ± 0.063 | 22 ± 7 | 2.79 ± 0.42 | 42.32 ± 1.52 | 15.51 ± 3.11 | 19.37 ± 1.38 | 0.11 ± 0.03 | 0.90 ± 0.51 |
|  | drought | 0.099 ± 0.079 | 0.329 ± 0.174 | 0.167 ± 0.055 | 0.462 ± 0.099 | 43 ± 26 | 2.84 ± 0.10 | 43.03 ± 0.28 | 15.17 ± 0.52 | 21.31 ± 5.02 | 0.29 ± 0.17 | 3.66 ± 2.63 |
| *Lc* | ambient | 0.753 ± 0.203 | 0.187 ± 0.066 | 0.177 ± 0.033 | 0.376 ± 0.028 | 52 ± 6 | 2.62 ± 0.20 | 44.87 ± 0.43 | 17.17 ± 1.19 | 19.43 ± 2.52 | 1.79 ± 1.25 | 2.35 ± 1.21 |
|  | drought | 0.282 ± 0.113 | 0.249 ± 0.147 | 0.175 ± 0.041 | 0.291 ± 0.025 | 89 ± 12 | 2.49 ± 0.14 | 44.96 ± 0.35 | 18.07 ± 0.98 | 22.04 ± 4.81 | 0.65 ± 0.43 | 2.92 ± 0.74 |
| *Tp* | ambient | 0.702 ± 0.188 | 0.189 ± 0.041 | 0.198 ± 0.064 | 0.275 ± 0.017 | 96 ± 41 | 2.86 ± 0.21 | 44.83 ± 0.50 | 15.72 ± 1.14 | 18.76 ± 3.95 | 0.93 ± 0.42 | 1.43 ± 0.73 |
|  | drought | 0.233 ± 0.106 | 0.193 ± 0.091 | 0.167 ± 0.033 | 0.273 ± 0.060 | 117 ± 49 | 2.85 ± 0.19 | 43.62 ± 0.94 | 15.33 ± 0.95 | 23.95 ± 2.84 | 0.57 ± 0.40 | 4.65 ± 6.95 |
| *Tr* | ambient | 0.647 ± 0.235 | 0.159 ± 0.036 | 0.189 ± 0.020 | 0.278 ± 0.025 | 90 ± 17 | 2.93 ± 0.09 | 43.84 ± 0.97 | 14.99 ± 0.60 | 20.84 ± 2.55 | 1.66 ± 0.74 | 2.69 ± 0.95 |
|  | drought | 0.331 ± 0.169 | 0.188 ± 0.101 | 0.182 ± 0.029 | 0.259 ± 0.018 | 107 ± 20 | 2.57 ± 0.37 | 44.31 ± 0.39 | 17.47 ± 2.34 | 23.76 ± 3.56 | 0.87 ± 0.46 | 3.44 ± 2.70 |

**Table S4**

|  | **PC1** | | R^2^ adj. fixed: 0.83 R^2^ adj. random: 0.09 |  | **PC2** | | R^2^ adj. fixed: 0.08 R^2^ adj. random: 0.68 | |
| --- | --- | --- | --- | --- | --- | --- | --- | --- |
| **term** | DF | χ^2^ | *P*-value |  | DF | χ^2^ | | *P*-value |
| functional group (FG) | **2** | **81.639** | **<0.0001** |  | 2 | 1.787 | | 0.409 |
| watering treatment (WT) | **1** | **5.218** | **0.022** |  | **1** | **4.774** | | **0.029** |
| FG:WT | **2** | **8.230** | **0.016** |  | **2** | **5.986** | | **0.050** |

**Table S5:**

| Total plant-soil feedback | | | | | | | R^2^ adj. fixed: 0.21  R^2^ adj. random: 0.29 | |
| --- | --- | --- | --- | --- | --- | --- | --- | --- |
| model | PSF ~ PC1home * WT + PC2home * WT +  (1\|block) + (1\|species home/species away) | | | | | | | |
|  | **PC1** | | |  | **PC2** | | | |
| term | DF | χ^2^ | *P*-value |  | DF | χ^2^ | | *P*-value |
| location home (Loc H) | **1** | **10.362** | **0.001** |  | 1 | 0.359 | | 0.549 |
| watering treatment (WT) | 1 | 1.284 | 0.257 |  | 1 | 1.284 | | 0.257 |
| Loc H : WT | 1 | 2.325 | 0.127 |  | 1 | 0.958 | | 0.328 |
|  |  |  |  |  |  |  | |  |
| Specific plant-soil feedback | | | | | | | R^2^ adj. fixed: 0.01  R^2^ adj. random: 0.21 | |
| model | PSF ~ PC1home * PC1away * WT + PC2home * PC2away * WT + (1\|block) + (1\|species home/species away) | | | | | | | |
|  | **PC1** | | |  | **PC2** | | | |
| term | DF | χ^2^ | *P*-value |  | DF | χ^2^ | | *P*-value |
| location home (Loc H) | 1 | 1.659 | 0.198 |  | 1 | 0.085 | | 0.771 |
| location away (Loc A) | 1 | 0.071 | 0.790 |  | 1 | 1.180 | | 0.277 |
| watering treatment (WT) | 1 | 0.185 | 0.667 |  | 1 | 0.185 | | 0.667 |
| Loc H : Loc A | 1 | 0.554 | 0.457 |  | 1 | 0.424 | | 0.515 |
| Loc H : WT | **1** | **6.503** | **0.011** |  | 1 | 2.292 | | 0.130 |
| Loc A : WT | 1 | 0.045 | 0.832 |  | 1 | 0.047 | | 0.828 |
| Loc H : Loc A : WT | 1 | 0.952 | 0.329 |  | 1 | 2.163 | | 0.141 |


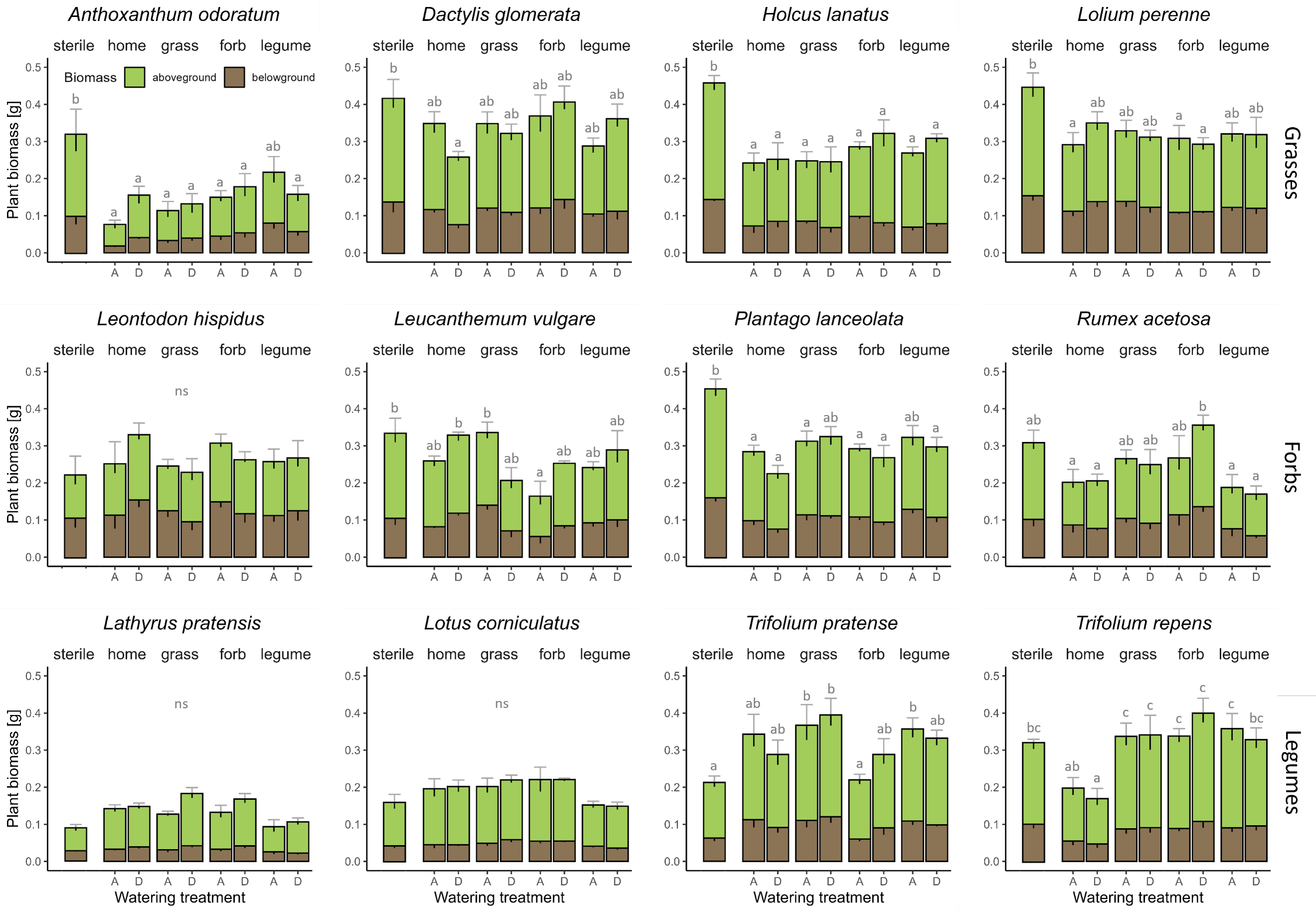


**Figure S1**


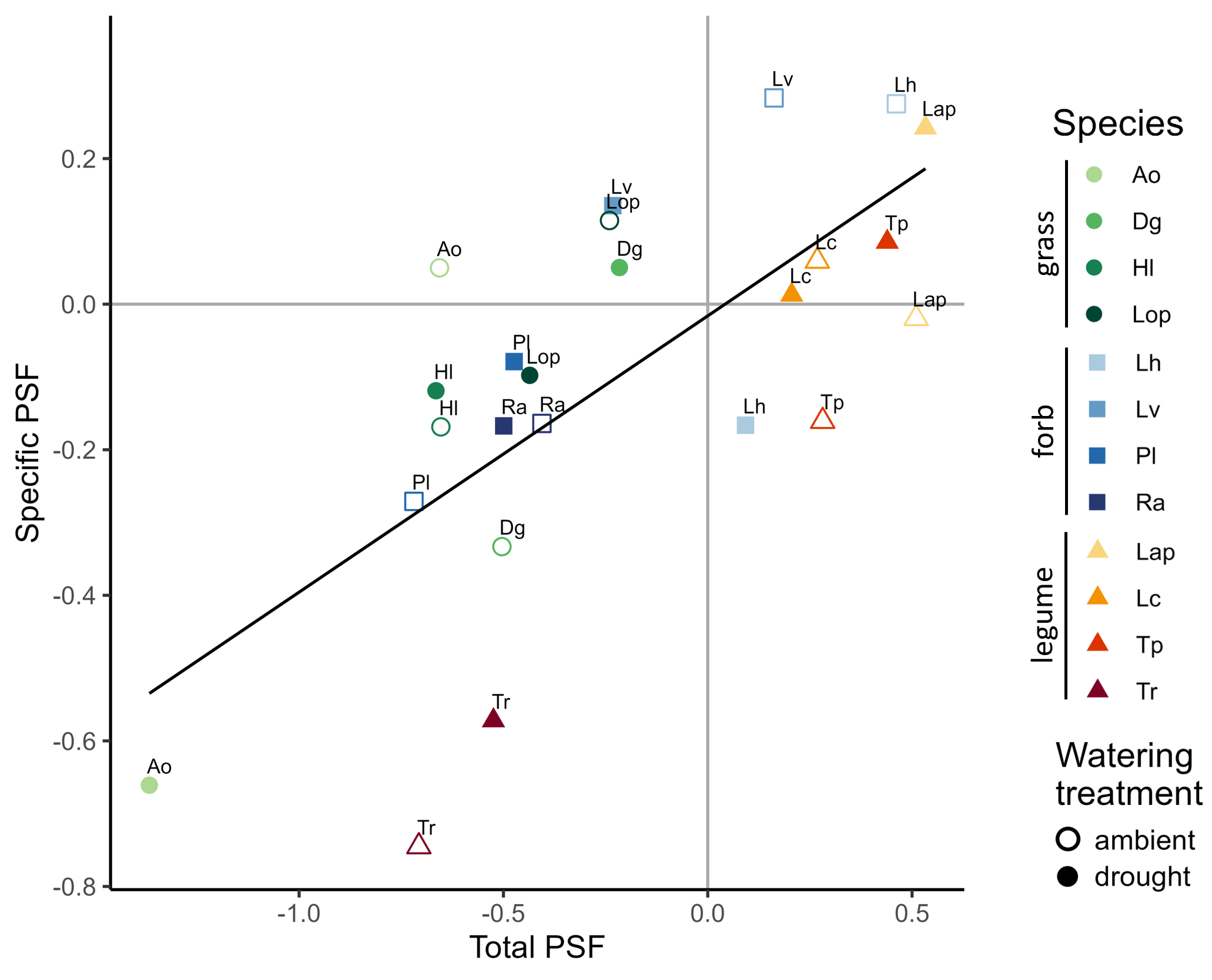


**Figure S2**

**
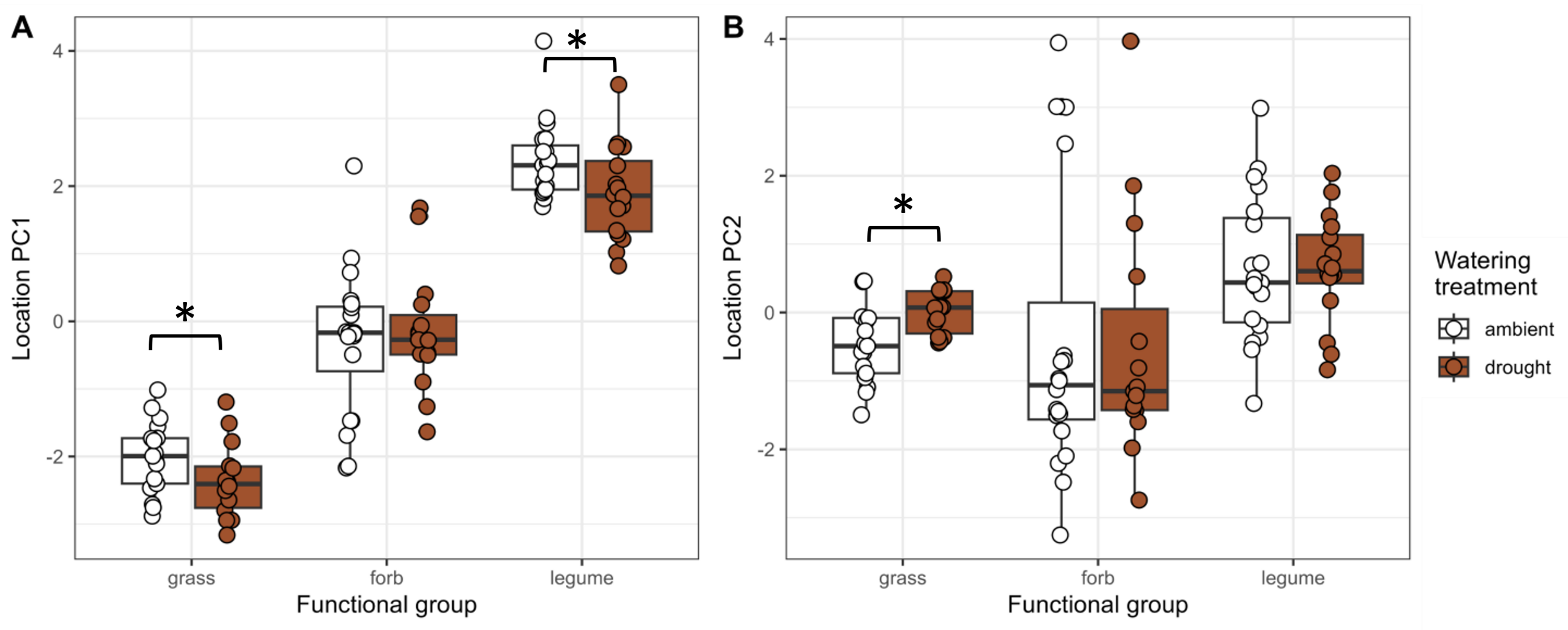
**

**Figure S3**


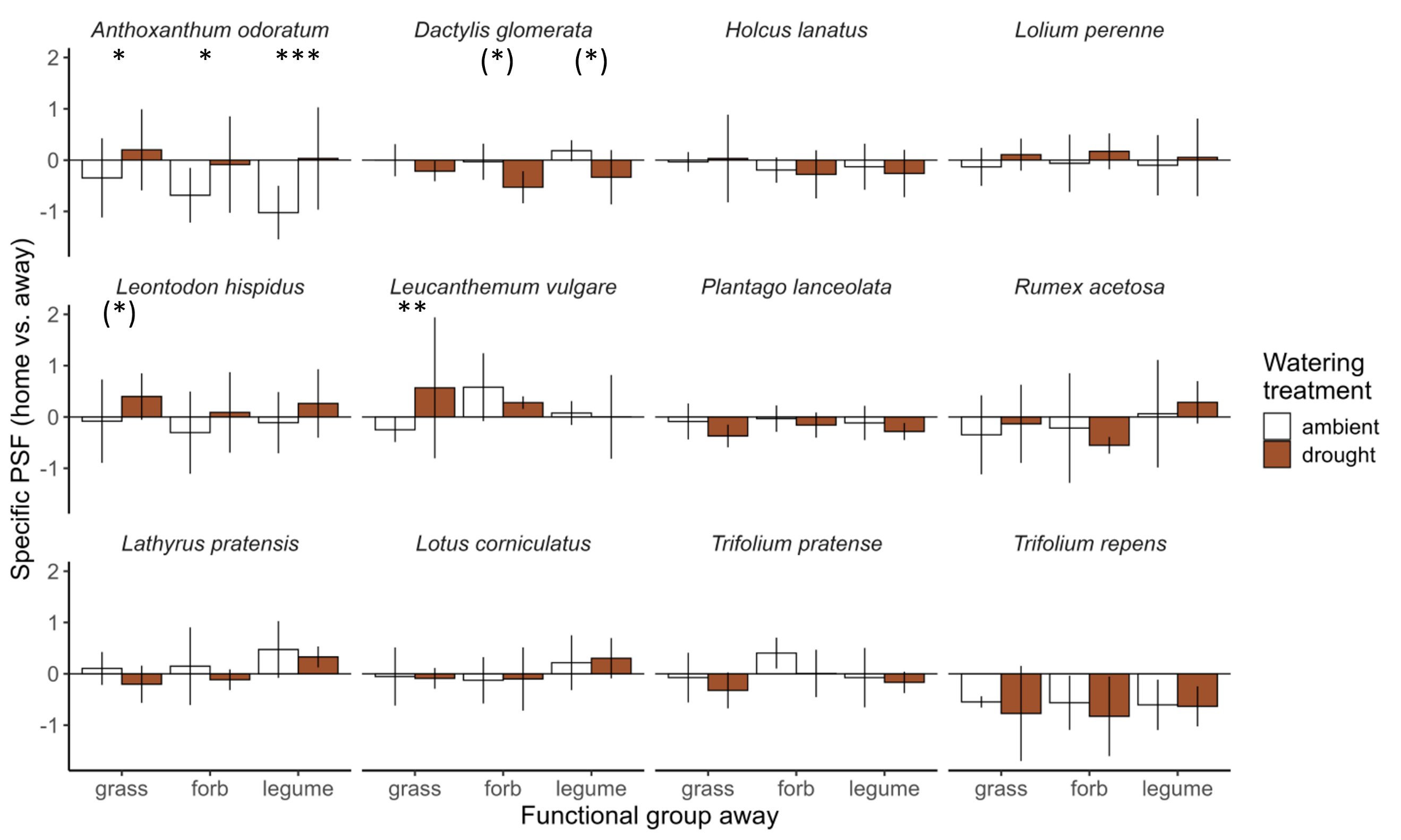


**Figure S4**


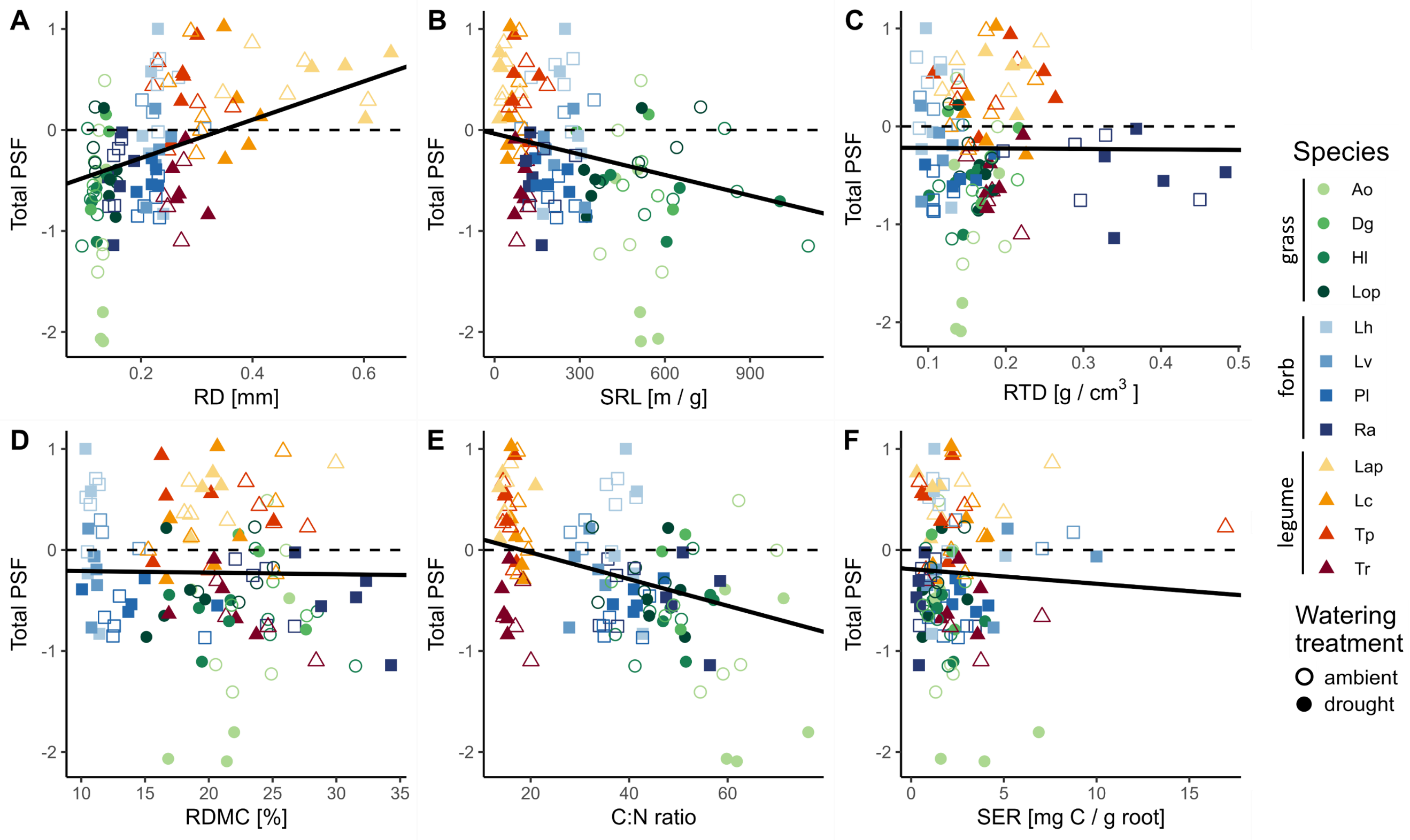


**Figure S5**


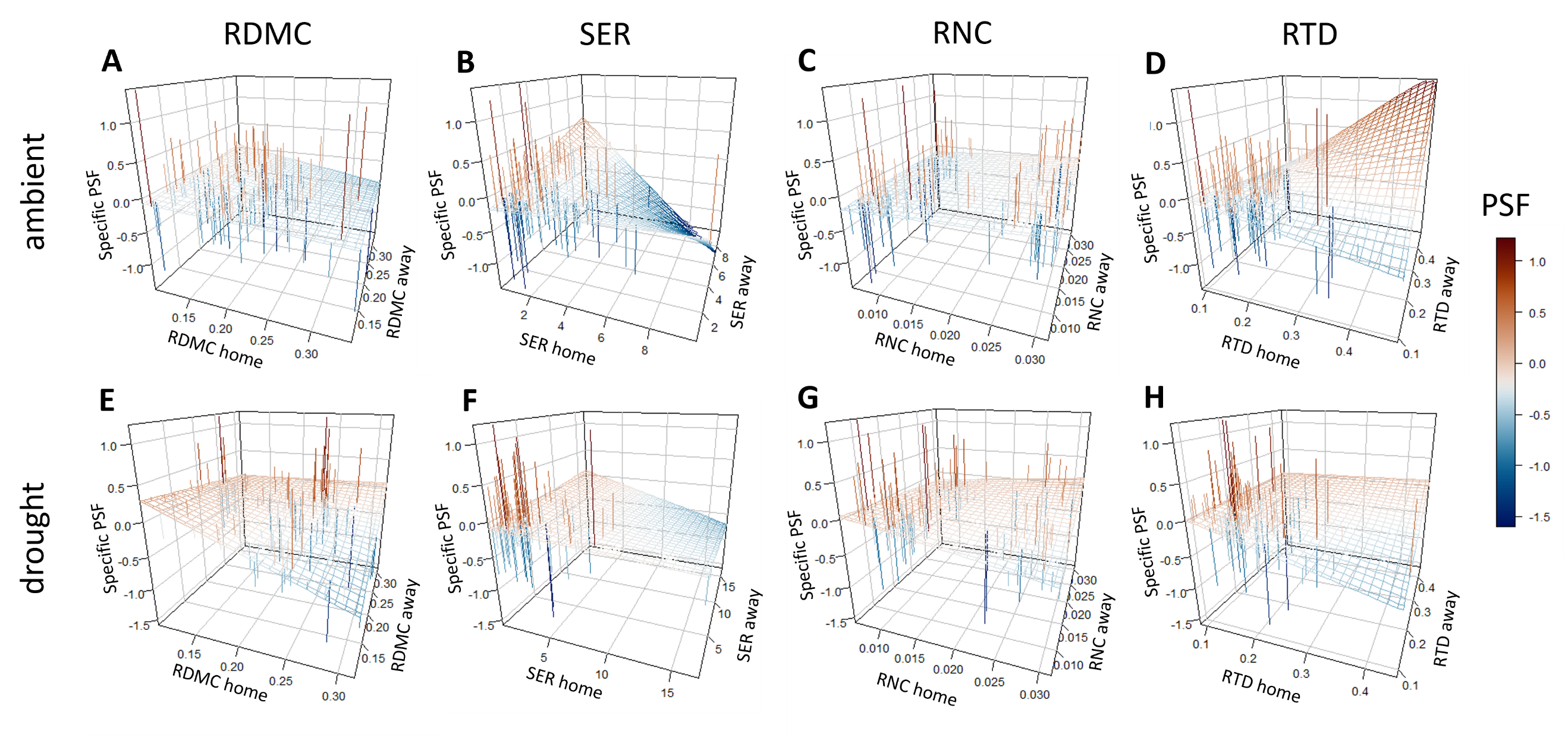


**Figure S6**
